# Hereditary cardiac amyloidosis associated with Pro24Ser transthyretin mutation: a case report

**DOI:** 10.1186/s13256-018-1931-5

**Published:** 2018-12-16

**Authors:** Hiroyuki Yamamoto, Toru Hashimoto, Shunji Kawamura, Michiaki Hiroe, Taro Yamashita, Yukio Ando, Tomoki Yokochi

**Affiliations:** 1 Department of Cardiology, Cardiovascular Center, Showa General Hospital, 8-1-1 Hanakoganei, Kodaira City, Tokyo 187-8510 Japan; 2 Department of Cardiovascular Medicine, Narita-Tomisato Tokushukai Hospital, Chiba, Japan; 3 Department of Pathology, Tokyo Women’s Medical University, Tokyo, Japan; 4 Department of Cardiology, Center Hospital of the National Center for Global Health and Medicine, Tokyo, Japan; 5 Department of Neurology, Graduate School of Medical Sciences, Kumamoto University, Kumamoto, Japan; 6 Department of Clinical Research, Chiba Tokushukai Hospital, Chiba, Japan

**Keywords:** Transthyretin, Cardiac amyloidosis, Hereditary ATTR amyloidosis

## Abstract

**Background:**

Transthyretin amyloidosis is a systemic disorder caused by extracellular deposition of insoluble amyloid fibrils in peripheral and autonomic nerves, heart, kidney, gastrointestinal tract, and other organs. Hereditary transthyretin amyloidosis is an autosomal dominant disease. More than 120 mutations have been reported in the transthyretin gene with considerable phenotypic heterogeneity and geographic diversity. Among them, a sporadic case of hereditary transthyretin amyloidosis with cardiac-predominant phenotype is very rare, progressive, and potentially fatal if left undiagnosed. However, a clinical diagnosis of cardiac amyloidosis still remains challenging due to non-specific symptoms, and less sensitivity and specificity of medical examinations.

**Case presentation:**

A 60-year-old Japanese man with a history of embolic stroke and hypertrophic cardiomyopathy visited our department for heart failure. The present case exhibited only cardiomyopathy without any clinical signs of systemic amyloidosis manifested as carpal tunnel syndrome, polyneuropathy, or autonomic dysfunction. An echocardiogram revealed severe asymmetric left ventricular hypertrophy, biatrial dilatation, pericardial effusion, and preserved left ventricular ejection fraction of 50% with severe diastolic dysfunction. Technetium pyrophosphate scintigraphy indicated marked diffuse myocardial uptake of technetium pyrophosphate, strongly suggesting transthyretin cardiac amyloidosis, which was firmly confirmed by a left ventricular endomyocardial biopsy. Genetic analysis demonstrated a transthyretin C70T (Pro24Ser) heterozygous mutation. Tafamidis, a transthyretin stabilizer, was started. His cardiac symptoms remained unchanged for 12 months.

**Conclusions:**

Here we report the case of a patient with hereditary cardiac amyloidosis associated with a Pro24Ser mutation in transthyretin, which is the first case reported in Japan. Technetium pyrophosphate scintigraphy was extremely useful for definitive diagnosis. Thus, we propose that the nuclear imaging technique should be taken into account even for an exploratory diagnosis of transthyretin cardiac amyloidosis.

## Background

Transthyretin (TTR) amyloidosis is a systemic disorder caused by extracellular deposition of insoluble amyloid fibrils in peripheral and autonomic nerves, heart, kidney, gastrointestinal tract, and other organs. A TTR gene product is one of the components of amyloid, which is a plasma protein predominantly produced in the liver, acting as a transporter of thyroxine and retinol-binding protein. Hereditary amyloid TTR (ATTR) amyloidosis is an autosomal dominant disease in which gene mutations lead to changes in the protein TTR [1].

More than 120 mutations have been reported in the *TTR* gene with considerable phenotypic heterogeneity and geographic diversity. A substitution of 30th valine by methionine (Val30Met) is the most common genetic variant found in endemic areas in Portugal, Sweden, and Japan [2]. The Val30Met variant induces progressive neurological symptoms, such as axonal sensory autonomic and motor neuropathy, whereas other mutations (Val122Ile, Thr60Ala, Leu111Met, and Ile68Leu) predominantly show infiltrative cardiomyopathy [3].

A sporadic case of hereditary ATTR cardiac amyloidosis is very rare, progressive, and potentially fatal if left undiagnosed; thus, early diagnosis is critical. However, the clinical diagnosis of cardiac amyloidosis remains challenging because neurological symptoms are less recognized in a sporadic case [4]. Therefore, development of an appropriate strategy to reach correct diagnosis of cardiac amyloidosis has been long overdue.

Here we report the case of a patient with cardiac amyloidosis associated with a single mutation in TTR, which is the first case in Japan. In addition, we propose a new diagnostic strategy of cardiac amyloidosis, which may contribute to establishing an early and differential diagnosis of this rare cardiac disease.

## Case presentation

**Fig. 1:**
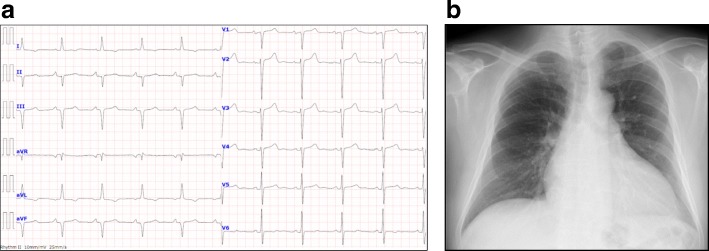
**a** A 12-lead electrocardiogram. **b** Chest X-ray

**Fig. 2:**
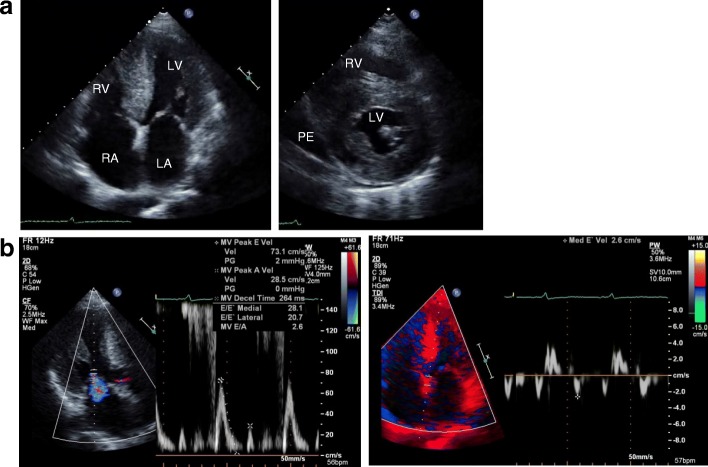
Transthoracic echocardiography in apical four chamber (**a**, *left panel*) and short axis view (*right*). Transmitral flow revealed an early mitral inflow to late filling velocity ratio of 2.6 (**b**, *left panel*). Tissue Doppler imaging of the mitral annulus (*right*). *LA* left atrium, *LV* left ventricle, *PE* pericardial effusion, *RA* right atrium, *RV* right ventricle

**Fig. 3:**
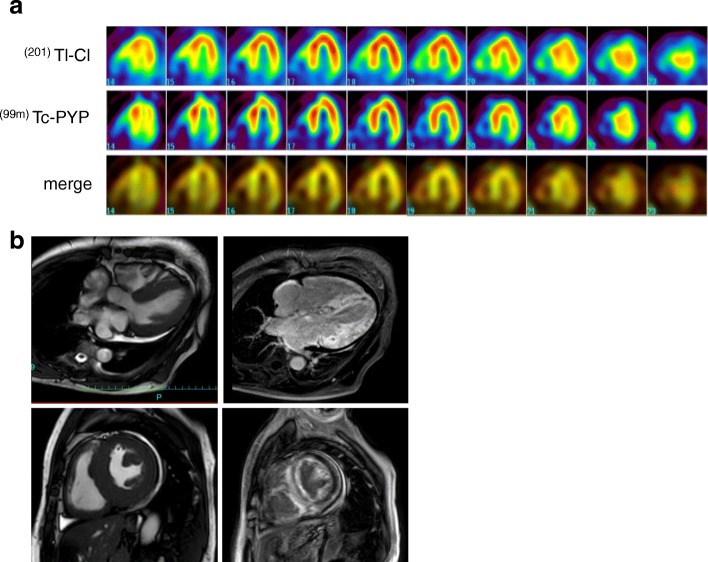
**a** Horizontal long-axis images of cardiac dual-isotope technique (thallium chloride and technetium pyrophosphate). thallium chloride was used for myocardial perfusion as a control. **b** The late gadolinium enhancement images in the horizontal long-axis (*upper panels*) and short-axis views (*lower panels*). ^*99m*^*Tc-PYP* technetium pyrophosphate, ^*201*^*Tl-Cl* thallium chloride

**Fig. 4:**
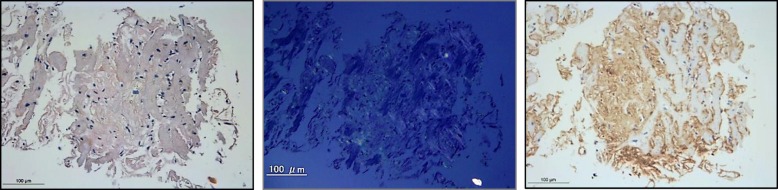
Endomyocardial biopsy images. Congo red staining showed amyloid deposits within the tissue (*left*), with apple-green birefringence under polarized light (*center*). Immunostaining against transthyretin was positive (*right*). *Bars*, 100 μm

**Fig. 5:**
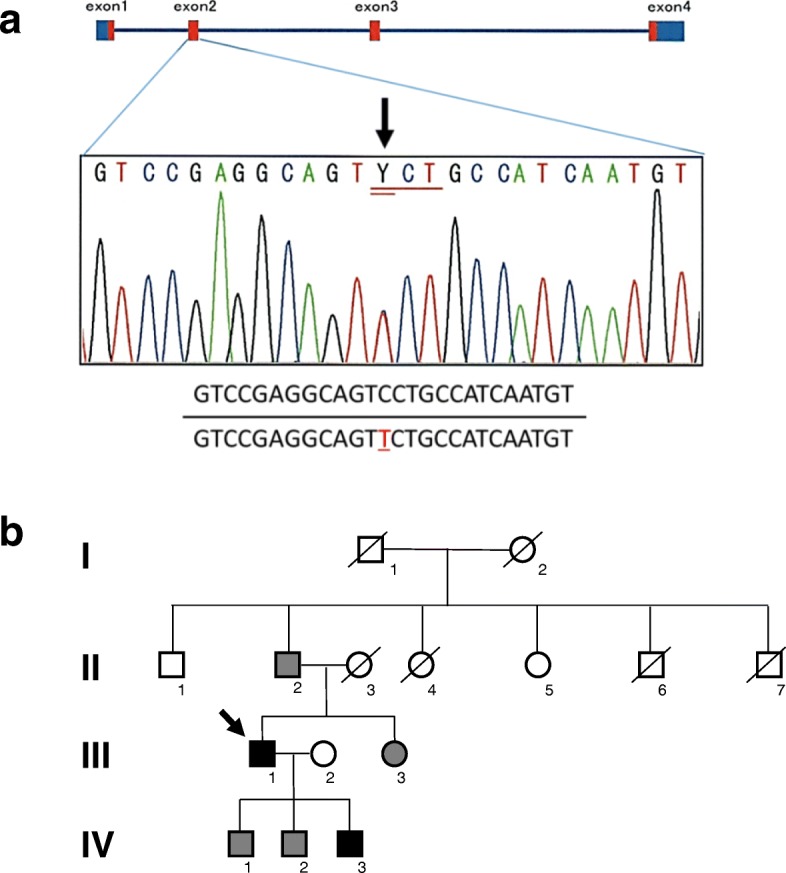
**a** DNA sequencing of transthyretin indicates mutation in exon2 with a nucleotide substitution at 70th (C70T), resulting in Pro24Ser variant of the transthyretin protein. **b** Genotyping of transthyretin in family members of the patient. *Arrow* indicates the proband. Individuals with heterozygous Pro24Ser mutation (*filled symbols*); wild-type (*shaded*); not tested (*open*)

A 60-year-old Japanese man visited our department for heart failure. He did not smoke tobacco; he had two histories of cardioembolic cerebral infarction at ages 47 and 59. Also, he had hypertrophic cardiomyopathy at age 58, but had no coronary risk factors including hypertension. His blood pressure was 107/72 mmHg with a heart rate of 60 beats per minute. He had a grade 2/6 systolic murmur and mild pretibial edema. Carpal tunnel syndrome, polyneuropathy, and autonomic dysfunction were unremarkable. An electrocardiogram showed normal sinus rhythm with QS waves in inferior leads, and with low QRS voltages in leads V1 to V4 (Fig. 1a). A chest X-ray showed cardiomegaly (Fig. 1b). An echocardiogram demonstrated severe asymmetric left ventricular hypertrophy (LVH; the interventricular septum and the posterior wall were 13 mm and 16 mm, respectively), biatrial dilatation, pericardial effusion, and preserved left ventricular ejection fraction of 50% (Fig. 2a). Increased right ventricular wall thickness was also seen. There was grade III diastolic dysfunction (Fig. 2b). The unexplained LVH led us to suspect cardiac amyloidosis. Technetium pyrophosphate (^99m^Tc-PYP) scintigraphy indicated marked diffuse myocardial uptake of ^99m^Tc-PYP (Fig. 3a), which strongly suggested TTR cardiac amyloidosis. In addition, cardiac magnetic resonance imaging revealed wide-spreading transmural late gadolinium enhancement at the ventricular and atrial walls, also supporting this notion (Fig. 3b). A left ventricular endomyocardial biopsy confirmed TTR-related amyloid deposits (Fig. 4). DNA sequence analysis demonstrated a TTR C70T (Pro24Ser) heterozygous mutation (Fig. 5a). Therefore, we assume that the Pro24Ser mutation is responsible for cardiac amyloidosis. Further genotyping of TTR of the family members of our patient revealed that his third son has the identical mutation (Fig. 5b), while he showed no clinical signs. Our patient was ineligible for heart transplantation due to his age (over 60) and renal dysfunction; thus, a combined usage of diuretics and tafamidis, a TTR stabilizer, was administered. His cardiac symptoms remained unchanged for 12 months.

## Discussion

In this report, we described the first case of hereditary cardiac amyloidosis associated with a Pro24Ser TTR mutation in Japan. Also, we demonstrated that nuclear imaging is effectively useful for definitive diagnosis of cardiac amyloidosis.

A rare case of hereditary amyloidosis associated with the TTR mutation (Pro24Ser) has been reported previously [5]. All siblings of the single family had similar, late-onset symptoms of systemic amyloidosis manifested as carpal tunnel syndrome, polyneuropathy, autonomic dysfunction, and cardiomyopathy. Note that these phenotypes were located at both the nervous system and cardiac muscle. On the other hand, the patient described in this report exhibited an isolated cardiac-predominant phenotype despite having the same mutation. In general, mutations that cause genetic disease strongly correlate with many aspects of phenotype, including onset, location, and the degree of progression. It has been reported, however, that there are several cases exhibiting inconsistency in terms of genotype-phenotype correlation. For instance, TTR Val30Met yields a variety of phenotypes including age of onset, severity, genetic penetrance, and regional specificity [1]. In Japan and Portugal, the TTR Val30Met mutation is frequently found in endemic familial history, presenting neurological-predominant phenotypes that begin at an early age. In contrast, the mutation found in Sweden is rather sporadic, and is associated with late onset and cardiac-predominant phenotypes [6]. This heterogeneity may be explained by several possibilities, such as epigenetic configuration around *TTR* gene, additional mutation in another gene not yet identified, or transcriptional regulation mediated by non-coding RNA [7, 8].

A definitive diagnosis of cardiac amyloidosis is significantly difficult because LVH is a common symptom in hypertension, aortic stenosis, and hypertrophic cardiomyopathy [9]. This case had been considered to have hypertrophic cardiomyopathy because of asymmetric hypertrophy. He had several episodes of cardioembolic infarction despite normal sinus rhythm, followed by heart failure. Intrinsic atrial dysfunction induced by amyloid deposition to the heart muscle may result in atrial thrombi [10, 11]. These clinical signs may provide a critical clue to early diagnosis of cardiac amyloidosis. Therefore, cardiac amyloidosis should be taken into account when LVH is accompanied by embolic stroke or heart failure.

Infiltration of the heart by amyloidogenic proteins induces several types of cardiac amyloidosis, including amyloid immunoglobulin light-chain (AL) amyloidosis, hereditary ATTR amyloidosis, and wild-type ATTR amyloidosis. Limited specificity in diagnostic techniques and poor sensitivity of noncardiac biopsy or abdominal fat aspiration may result in delayed diagnosis [12]. Recently, a nuclear imaging technique employing ^99m^Tc-PYP has been described as a reliable diagnostic tool for ATTR cardiac amyloidosis, which is distinguished from AL amyloidosis with high specificity, and cardiac TTR deposition can be detected at an early asymptomatic stage [13, 14]. In fact, we demonstrated in this report that cardiac radioisotope examination was very effective in the diagnosis of hereditary ATTR cardiac amyloidosis. This is the first case of hereditary cardiac amyloidosis associated with a TTR mutation (Pro24Ser) in Japan. We propose that an exploratory diagnosis utilizing nuclear imaging is highly reliable to identify ATTR cardiac amyloidosis.

## Conclusions

We report a case of isolated cardiac amyloidosis associated with a Pro24Ser mutation in TTR, which is the first case reported in Japan. We demonstrated that ^99m^Tc-PYP scintigraphy is extremely useful for definitive diagnosis of this rare disease.

## Abbreviations

^99m^Tc-PYP: Technetium pyrophosphate
AL: Amyloid immunoglobulin light-chain
ATTR: Amyloid transthyretin
LVH: Left ventricular hypertrophy
TTR: Transthyretin
